# The independent predictive effect of insomnia symptoms before high-altitude exposure on acute mountain sickness: an observational study of healthy volunteers at 2726 m

**DOI:** 10.3389/fpsyt.2026.1765494

**Published:** 2026-03-09

**Authors:** Shaofei Hou, Jiahao Jiang, Yating Huang, Yujia Liang, Xinyu Zhou, Lun Li

**Affiliations:** School of Physical Education, China University of Geosciences, Wuhan, China

**Keywords:** acute mountain sickness, athens insomnia scale, high altitudes, highly educated individuals, symptoms of insomnia

## Abstract

**Objective:**

To examine the independent predictive role of symptoms of insomnia on the risk of acute mountain sickness (AMS) in healthy volunteers during a short-term extreme-altitude mountaineering activity.

**Methods:**

Fifteen healthy volunteers were recruited for a 3-day mountaineering expedition with a target altitude of 5,396 meters. Baseline symptoms of insomnia were assessed one week before departure at low altitude (Wuhan, 23 m) using the Athens Insomnia Scale (AIS). During the 3-day ascent, high-altitude insomnia symptoms and AMS symptoms were evaluated daily using the AIS and the 2018 Lake Louise Score (LLS), respectively. The primary analysis for AMS, based on LLS assessment (Day 2 post-arrival), was conducted at an altitude of 2726 m. Univariate and multivariate logistic regression analyses were conducted to examine the association between AIS scores and AMS.

**Results:**

The mean baseline AIS score of participants was 6.9, and the mean high-altitude AIS score was 10.7. During the expedition, AMS (defined as LLS≥3) occurred in 10 participants, with a mean LLS score of 4.3 among AMS cases. Univariate analysis showed that both baseline AIS score (OR = 2.994,95%CI:1.059–8.459, p<0.05) and high-altitude AIS score (OR = 3.901,95% CI: 1.124–13.544, p<0.05) were significantly positively associated with AMS risk. In multivariate analysis, after adjusting for age and gender, baseline AIS score remained an independent predictor of AMS (OR = 3.074, 95% CI:1.023–8.638, p<0.05), whereas the high-altitude AIS score did not yield a stable estimate due to complete separation in the model. Univariate analysis indicated that ΔAIS (change in AIS score) did not show a significant effect on AMS (p>0.05).

**Conclusion:**

Baseline AIS scores serve as an independent predictor of AMS, supporting the incorporation of sleep assessment into pre-ascent. health screening for high-altitude mountaineering. However, this finding is derived from a small sample within a specific cohort; its predictive utility and generalizability must be independently validated in future studies with larger and more diverse populations.

## 1 Introduction

Acute Mountain Sickness (AMS) is a common clinical syndrome occurring after rapid ascent to altitudes above 2,500 meters, primarily attributed to hypoxic exposure. Its core symptoms include headache, dizziness, nausea, fatigue, among others, and in severe cases, it may progress to high-altitude cerebral edema, directly threatening the lives of mountaineers (1, 2). In addition to these core diagnostic symptoms, a subset of patients may also experience sleep disturbances following high-altitude exposure. While sleep disturbances induced by the high-altitude environment such as periodic breathing and sleep fragmentation-constitute an independent physiological burden, which may contribute to the development of acute mountain sickness (AMS) (3). Research indicates that, compared to passive ascent, active ascent significantly accelerates the onset timeline of Acute Mountain Sickness (AMS) and may increase its early severity (4, 5). With the increasing popularity of high-altitude scientific research, mountaineering expeditions, and plateau tourism, the frequency with which highly educated individuals (e.g., university staff, students, and researchers) travel to very high altitudes(defined as >3,500 meters) for work or scientific investigation has risen significantly (6, 7). While this demographic typically possesses a high level of health literacy, modern lifestyle factors such as work-related stress and irregular sleep patterns, which often accompany their profiles, may predispose them to distinct physiological and psychological response patterns in high-altitude environments (8, 9). These factors could subsequently influence their susceptibility to AMS.

Existing research has established that the incidence of AMS is closely associated with physiological factors including ascent rate, prior high-altitude exposure, hypoxic ventilatory response, and decreased oxygen saturation (10, 11). In recent years, scholarly attention has turned to the role of sleep disorders in the pathogenesis of AMS. The high-altitude environment predisposes individuals to difficulties falling asleep, nocturnal awakenings, and reduced sleep quality. These sleep issues may not only be prodromal symptoms of AMS but could also exacerbate hypoxic stress and impair cognitive and emotional states, potentially creating a vicious “sleep-AMS” cycle (2, 12). A study conducted at 3,700 meters in China demonstrated a significant positive correlation between insomnia symptoms and AMS prevalence, identifying a higher score on the Athens Insomnia Scale (AIS) as an independent predictor for AMS (13). Similarly, Tang et al. found anxiety and poor sleep quality to be independent risk factors for AMS among young Chinese males (14). However, previous studies have largely failed to clarify the causal relationship between symptoms of insomnia and AMS, as the high-altitude environment itself can trigger sleep problems. Therefore, exploring whether pre-existing sleep insufficiency or disturbance before high-altitude exposure influences the body’s subsequent tolerance to such exposure represents a more forward-looking yet under-investigated question. Research has suggested that examining pre-ascent sleep conditions as a predictor of AMS is an important direction for future studies (15). An experimental study conducted in a normobaric hypoxic chamber offers preliminary insight: severe sleep restriction (<3 hours) prior to normobaric hypoxic exposure (simulating 3500 m) led to a significant increase in subjects’ Lake Louise Scores (LLS), although the mean value did not reach the clinical diagnostic threshold for AMS, and symptoms were primarily concentrated in the domain of fatigue (16). This suggests that baseline sleep deprivation may exacerbate the symptom burden under hypoxia, and sleep quality could be an important susceptibility marker independent of on-site high-altitude factors.

Based on the above background, this study proposes the following hypothesis: both pre-ascent baseline symptoms of insomnia (AIS score) and newly-onset or aggravated symptoms of insomnia during the ascent can independently predict the occurrence of acute mountain sickness (AMS). To this end, This study aims, through a prospective observational design, to achieve the following objectives: 1. Primary Objective: To assess the predictive value of pre-ascent baseline AIS scores for the occurrence of AMS at the 2726 m base camp in healthy volunteers during an ascent to the target altitude of 5396 m; 2. Secondary Objective: To analyze the predictive value of AIS scores for AMS during the stay at the 2726 m base camp, and to examine the impact of AIS score changes (ΔAIS) on AMS risk. By advancing the sleep assessment window to before high-altitude exposure, this study aims to provide novel temporal evidence for understanding the role of sleep in susceptibility to AMS.

## 2 Methods

### 2.1 Research design and procedure

**Figure 1:**
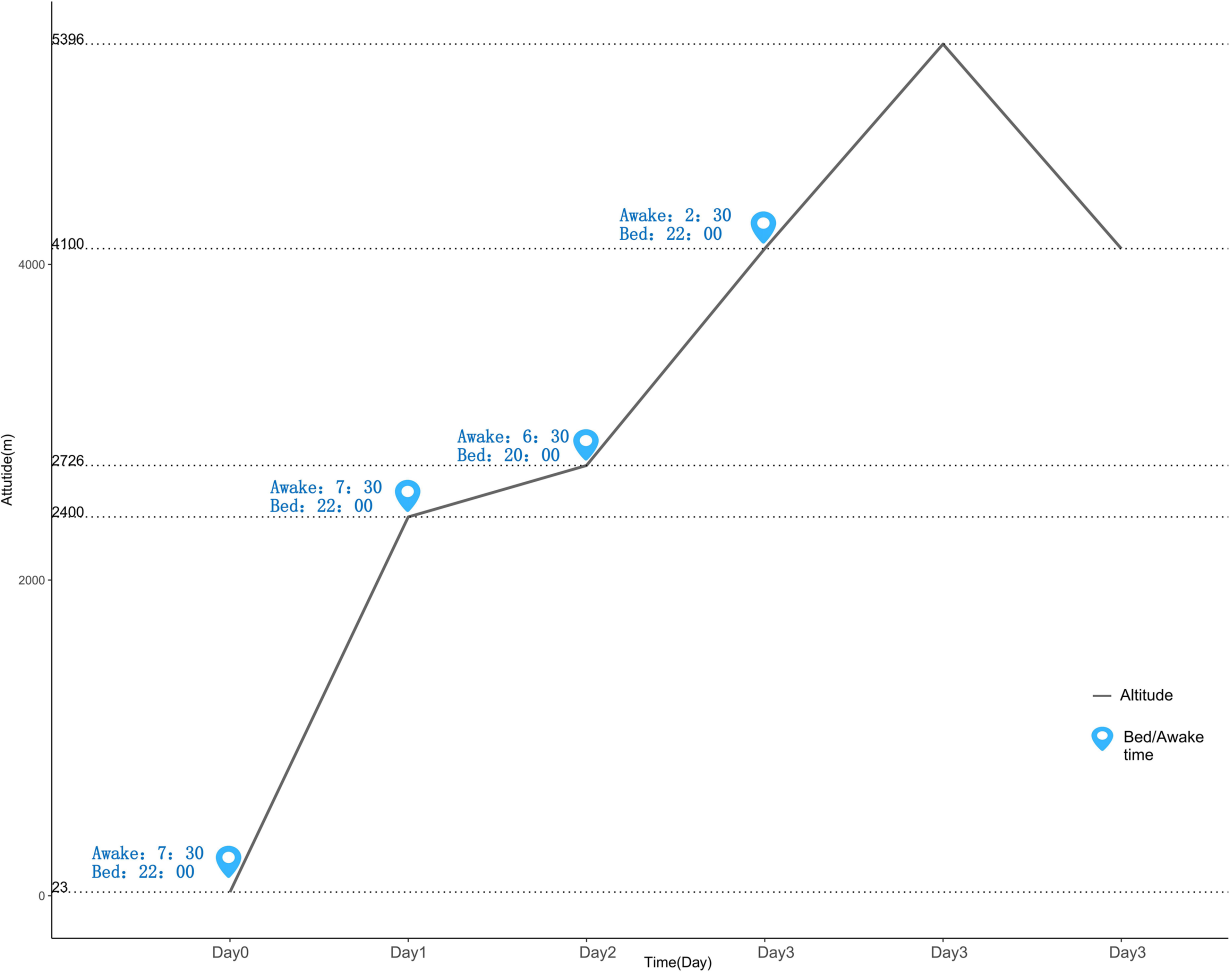
Elevation change.

This study employed an observational cohort design conducted from July 15 to 17, 2025, in the Haba Snow Mountain area of Yunnan Province, China. During the week prior to arriving at the study starting point (Lijiang), all participants were advised to follow a standardized preparation protocol: 1. Maintain their regular, habitual sleep-wake cycles and avoid staying up late; 2. Refrain from alcohol and caffeinated beverages; 3. Avoid using any medications that could affect sleep, hydration status, or altitude illness. Fifteen lowland participants completed baseline assessments using the Athens Insomnia Scale (AIS) one week prior to departure in Wuhan (23 meters above sea level) to evaluate baseline sleep conditions. The detailed procedure during high-altitude exposure is illustrated in **Figure 1**: On day one, the team departed by vehicle from Lijiang (2400 m) at 13:00 and arrived at Haba Village (2726 m) at 15:00 for logistical preparations, staying overnight at a local guesthouse. On day two, the team set out on a light hike from Haba Village at 07:00 and reached base camp (4100 m) at 15:50, where they stayed in tents. On day three, the ascent to the summit (5396 m) began at 03:00 from base camp, followed by descent and return. Throughout the expedition, camp setup, cooking, and other logistical tasks were managed by dedicated personnel to ensure team efficiency and safety.

All measurements were administered by trained investigators in a quiet environment 30 minutes after waking each morning (AIS and LLS on the summit day were assessed after returning to the base camp at 15:00). The symptoms of AMS, particularly headache and fatigue, can fluctuate throughout the day due to factors such as physical activity, dehydration, and light exposure. Conducting assessments at a relatively fixed time in the early morning, before the commencement of major daily activities, can minimize the influence of these diurnal confounding factors on the self-reporting of symptoms, thereby ensuring comparability of LLS scores across different days (17, 18). AIS scores were collected via mobile questionnaires, while LLS scores were recorded using paper-based forms. Participants remained seated during the assessments. Throughout the climb, all participants followed a unified schedule (lights-out at 22:00, wake-up at 07:30). Meals were provided collectively, with a daily water intake of at least 3 liters. The active ascent beyond Haba Village was completed via light hiking, with individual loads ranging approximately from 5 to 8 kg.

### 2.2 Research subjects

The sample size of this study was primarily determined by the team scale of this mountaineering expedition, aiming for exploratory observation and descriptive analysis. Fifteen lowland participants from China University of Geosciences (Wuhan) voluntarily joined the climbing activity and were assessed. All were graduate students, including three doctoral candidates and twelve master’s students (19). Prior to the expedition, all participants underwent physical examinations at designated hospitals. Only healthy, non-smoking, medication-free, and previously unmeasured climbers were included in the study. For baseline sleep assessment, the Athens Insomnia Scale (AIS) was used to screen participants’ sleep status. As shown in **Table 1**, the mean age of participants was 28 ± 8 years, with an average weight of 71.3 ± 11.5 kg and an average height of 176.3 ± 10.7 cm. To ensure that participants possessed the basic physical fitness required for the climb and could complete it safely with due regard to personal safety, a five-week physical training program was implemented before the expedition. Each session included 8 km running/trail running and core strength training, conducted three times per week, lasting 1–2 hours per session. For acclimatization to the mountain environment, a 12 km load-carrying hike was conducted one week before departure. The study protocol was approved by the Ethics Committee of China University of Geosciences (Approval No.: CUG2025-06-07), and written informed consent was obtained from all participants.

**Table 1:** Demographic characteristics of the participants.

Variable	All (n=15)	Male (n=8)	Female (n=7)
Age (year)	28 ± 8	29± 9	27± 6
Height (cm)	176.3 ± 10.7	184.8 ± 5.7	166.6 ± 6.6
Weight (kg)	71.3 ± 11.5	80.8 ± 7.1	60.4 ± 4.3
BMI (kg/m²)	22.9 ± 2.5	23.6 ± 1.6	21.8 ± 1.3
Having been to high altitude in recent 3 months	0	0	0
Drug	0	0	0

High altitude is defined as altitude ≥2500 meters. The value is expressed as mean ± standard deviation.

### 2.3 Symptom scores

#### 2.3.1 Athens insomnia scale

The Chinese version of the Athens Insomnia Scale (AIS) was used as the standardized instrument for assessing sleep quality (20, 21). In this study, we adapted the standard AIS instructions, asking participants to answer based on their experience over the past week (i.e., the 7 nights prior to the assessment day). This scale, aligned with core insomnia criteria from the International Classification of Sleep Disorders and the Chinese Guidelines for Adult Insomnia Diagnosis and Treatment, consists of 8 self-rated items. These items cover sleep induction, nocturnal awakenings, early morning awakening, total sleep time, sleep quality, daytime mood, daytime physical functioning, and daytime sleepiness. Each item is scored from 0 (no difficulty) to 3 (severe difficulty), yielding a total score ranging from 0 to 24. For this study, an AIS total score ≥ 6 was defined as indicating clinically significant insomnia symptoms, with severity categorized as mild (6–9), moderate (10–15), or severe (16–24) (22, 23). The scale typically asks participants to answer based on their sleep in the past week (24). The scale demonstrated internal consistency coefficients (Cronbach’s α) ranging from 0.81 to 0.86.

#### 2.3.2 Acute mountain sickness

The 2018 revised Lake Louise Scale was employed as the core screening and diagnostic tool for AMS (25, 26). According to international consensus, a diagnosis of AMS requires a total LLS score ≥ 3 in the presence of headache. The scale comprises four items: headache, gastrointestinal symptoms (nausea/vomiting), fatigue/weakness, and dizziness/light-headedness, each scored from 0 (none) to 3 (severe). The total score ranges from 0 to 12, with AMS severity classified as mild (3–5), moderate (6-9), or severe (10-12). It is noteworthy that despite the removal of sleep disturbance from the 2018 revised Lake Louise Scoring System, sleep disturbances continue to contribute to the diagnosis of acute mountain sickness (AMS) (27). All LLS assessments were strictly conducted at altitudes ≥ 2,500 meters to ensure specificity to high-altitude exposure and to exclude confounding factors present at lower elevations (28). The internal consistency coefficient (Cronbach’s α) for the LLS in this context was 0.84.

#### 2.3.3 Data analysis

As multiple studies have established that AMS symptoms typically onset within 6 to 12 hours of arrival at high altitude and peak within 24 hours (29, 30), the data collected at the 2726m base camp on the second day were selected for the main analysis. Specifically, the AMS status for each participant was determined using the LLS score assessed that morning in conjunction with the presence of headache. This AMS outcome was then analyzed in relation to the high-altitude AIS score obtained on the same day.

Data analysis was performed using SPSS version 27.0 (IBM, Chicago, IL, USA). Variables were coded as follows: the presence of Acute Mountain Sickness (AMS) served as the dependent variable (yes=1, no=0). Independent variables included the baseline and high-altitude AIS scores entered as continuous variables, while age (≥40 years=1, <40 years=0) and gender (male=1, female=0) were included as categorical variables. This study employed logistic regression models to analyze the relationship between AIS scores and the risk of AMS occurrence. First, univariate logistic regression was conducted to examine the independent effects of baseline AIS scores and high-altitude AIS scores, respectively (31, 32). To control for potential confounding factors, multivariate logistic regression was further performed by incorporating age and gender into the model to adjust for confounding effects (33). The significance level α was set at 0.05. An odds ratio (OR) >1 with a 95% confidence interval (CI) excluding 1 was considered indicative of a statistically significant risk factor for AMS. To explore more comprehensively the relationship between dynamic changes in sleep quality and the risk of acute mountain sickness (AMS), we calculated the difference between each participant’s high-altitude AIS score and their baseline AIS score (ΔAIS = AIS high altitude − AIS baseline). This ΔAIS metric was used to quantify the acute deterioration in sleep quality from plain to high-altitude environments. Subsequently, a univariate logistic regression model was applied to analyze the association between ΔAIS and the risk of AMS occurrence.

## 4 Results

### 4.1 LLS and AIS

At baseline, nine participants presented with mild insomnia symptoms (AIS score ≥ 6), while six had no insomnia (AIS score < 6). Continuous monitoring using the Athens Insomnia Scale and the Lake Louise Score during the three-day ascent revealed that, as shown in **Table 2**, on the second day, 10 participants developed acute mountain sickness. Among these, 9 participants had mild AMS (LLS score 3-5), and 1 participant had severe AMS (LLS score 11). On the third day of the itinerary, 10 participants developed acute mountain sickness, with a significant increase in the mean LLS score, and the highest LLS score reaching 12. During the summit push on Day 3, two participants met criteria for severe AMS. The research team immediately enacted the safety protocol, and they were escorted down to base camp by guides. After descending in altitude and resting for approximately 6–12 hours, both individuals’ AMS symptoms (LLS scores) improved significantly to a mild level.

**Table 2:** Descriptive statistics of LLS versus AIS scores on different study days.

Variable	Group	Day0 (Baseline, 23m)	Day1 (2400m)	Day2 (2726m)	Day3 (5396m)
AIS Score	All (N = 15)	6.9± 1.8	7.3 ± 1.8	10.7 ± 2.3	12.9 ± 2.0
	AMS+ (n=10)	7.7 ± 1.6	7.8 ± 1.9	11.8 ± 1.8	13.4 ± 1.9
	AMS- (n=5)	5.2 ± 1.1	6.2 ± 0.8	8.6 ± 1.5	12.0 ± 2.0
LLS Score	All (N = 15)	-	1.2± 1.7	3.5 ± 2.3	7.4 ± 3.0
	AMS+ (n=10)	-	1.3 ± 2.2	4.3 ± 2.5	8.7 ± 2.4
	AMS- (n=5)	-	0.8 ± 0.7	1.8 ± 0.4	4.8 ± 2.6

Data are presented as mean ± standard deviation. Since the baseline does not require the measurement of LLS scores, the LLS baseline data is represented by “-”.

### 4.2 Univariate analysis of AIS scores and AMS

As shown in **Table 3**, in the limited sample of this study (N = 15), the p-values for the baseline and high-altitude scores of the Athens Insomnia Scale (AIS) were 0.039 and 0.032, respectively, both below the threshold of 0.05. The odds ratio (OR) for the baseline AIS score was 2.994, with a 95% confidence interval (CI) ranging from 1.059 to 8.459 (34). Given the small sample size, the relatively wide confidence interval suggests some uncertainty in the effect estimate. Nonetheless, this result preliminarily indicates that each one-unit increase in the baseline AIS score was associated with approximately a 2.99-fold increase in the risk of developing acute mountain sickness (AMS). The fact that the confidence interval does not include 1 further supports the statistical significance of this finding. The odds ratio for the high-altitude AIS score was 3.901, with a 95% CI of 1.124 to 13.544. Similarly, the wide confidence interval calls for cautious interpretation of this estimate, suggesting that each one-unit increase in the high-altitude AIS score was associated with approximately a 3.90-fold increase in AMS risk. Compared with the baseline score, the higher OR observed for the high-altitude score may suggest that insomnia symptoms in high-altitude environments could have a greater influence on AMS risk.

**Table 3:** Univariate logistic regression analysis of risk factors for AMS.

Variable	AIS score	
	Baseline	High-altitude
β	1.096	1.361
Std. Error	0.530	0.635
Wald	4.281	4.596
df	1	1
p-value	0.039	0.032
Exp(B) [OR]	2.994	3.901
OR (95% CI)	1.059-8.459	1.124-13.544

### 4.3 Multivariate analysis and interaction test

As shown in **Table 4**, after controlling for age and gender, the baseline score of the Athens Insomnia Scale (AIS) showed a significance level of p = 0.046, with an odds ratio (OR) of 3.074 and a 95% confidence interval (CI) ranging from 1.023 to 8.638. This finding indicates that the baseline AIS score exerts an influence on acute mountain sickness (AMS) independently of age and gender (35, 36). It should be noted that due to the limited sample size, the statistical power of the model was constrained. In particular, the strong association between the AIS score at high altitude and AMS led to a phenomenon of “complete separation” in the model, which prevented reliable estimation of the effects of age and gender. Therefore, the non-significant p-values and the inestimable confidence intervals for the ORs of the high-altitude AIS score, age, and gender should be interpreted as a failure to detect significant effects within the current analytical framework and sample size, rather than as evidence against their potential associations.

**Table 4:** Multivariate logistic regression analysis of risk factors for AMS.

Variable	AIS score	Sex	Age
	baseline	baseline	baseline
β	1.123	-.430	-1.176
Std. Error	.562	1.606	3.297
Wald	3.988	.072	.127
df	1	1	1
p-value	.046	.789	.721
OR	3.074	.651	.309
OR (95% CI)	1.023-8.638	-.430-1.606	-1.176-3.297

### 4.4 Results of association analysis between changes in AIS score and AMS

As shown in **Table 5**, the regression coefficient for ΔAIS was 0.143, with a p-value of 0.573, which is well above the significance level of 0.05. The odds ratio (OR) was 1.154, with a 95% confidence interval ranging from 0.701 to 1.899. These results indicate that the change in AIS score did not demonstrate a statistically significant interaction with AMS in this sample. Based on the present data, ΔAIS cannot be considered an independent risk factor for the occurrence of AMS.

**Table 5:** Association analysis between changes in Athens Insomnia Scale scores and acute mountain sickness.

Variable	β	Std. error	Wald	df	P-value	OR	OR (95% CI)
ΔAIS	.143	.254	.318	1	.573	1.154	.701

## 5 Discussion

This study initially employed univariate logistic regression, revealing that both baseline AIS scores and high-altitude AIS scores were significantly positively associated with AMS incidence, with odds ratios (ORs) reaching 2.994 and 3.901, respectively. However, an important extended finding of this study is that an individual’s baseline insomnia symptoms (baseline AIS score) prior to arriving at high altitude also significantly predicted AMS risk. Our research further quantified the strength of this association: for each 1-point increase in the AIS score, the risk of AMS increased approximately 2 to 3 times. This strongly suggests that insomnia is not merely a concomitant symptom of AMS but may also be an important prodromal manifestation and pathogenic factor (37). The results indicate that the prevalence of acute mountain sickness (AMS) among participants in this study was slightly higher than in two other studies conducted at similar altitudes (13, 38). This may be because this specific group of graduate students already carries a degree of chronic sleep debt during their time at low altitude due to demanding academic and research responsibilities (39), rendering their brains more vulnerable when confronting the new stressor of acute hypoxia (40).

This study, along with several prior observations, suggests that sleep insufficiency at low altitude may be an independent susceptibility factor for AMS (15). Firstly, chronic sleep deprivation or poor sleep quality itself constitutes a persistent physiological stressor, which can lead to the accumulation of multiple physiological deficits in neuroendocrine, metabolic, and immune systems—that is, an increased “allostatic load” (41). When an individual enters high altitude carrying a higher allostatic load, their physiological reserve and compensatory capacity to cope with acute hypoxic stress are already diminished, making them more likely to surpass the threshold for AMS development. Secondly, sleep insufficiency can directly “pre-activate” or sensitize the body’s stress response systems (42); even short-term sleep loss can significantly elevate sympathetic nervous system tone and HPA-axis activity (15). When such individuals are exposed to the powerful new stressor of high-altitude hypoxia, their stress response may be amplified or accelerated (43). This parallels mechanisms observed in obstructive sleep apnea research, where intermittent hypoxia can trigger systemic inflammation, oxidative stress, and endothelial dysfunction (44). These amplified pathophysiological responses collectively exacerbate the risk of disturbances in cerebral blood flow regulation and vasogenic edema, thereby promoting the occurrence of AMS (45). The finding of a strong association between high-altitude AIS scores and AMS is highly consistent with conclusions from previous studies (46). Buguet et al. pointed out that alterations in sleep architecture represent one of the core physiological responses upon initial high-altitude exposure, and a bidirectional relationship exists between sleep quality and AMS occurrence (47). It is particularly noteworthy that the multivariate model incorporating the high-altitude AIS score encountered “complete separation”. Although this statistical phenomenon complicates the interpretation of results, from an epidemiological perspective, it inversely corroborates the extremely strong predictive power of the high-altitude AIS score, to the extent that in this limited sample, the effects of other variables were completely “drowned out” (48).

The association analysis between the change in AIS scores (ΔAIS) and AMS indicates that ΔAIS and AMS risk do not have a statistically significant effect, suggesting their contributions to AMS risk are relatively independent. This finding highlights the fundamental difference in the physiological origins of sleep disturbances at sea level versus high altitude. Baseline insomnia at sea level primarily reflects an individual’s long-term sleep fragility, chronic stress levels, or circadian traits, potentially underpinned by sustained hyperactivity of the hypothalamic-pituitary-adrenal axis, constituting a “background” risk for AMS (49). In stark contrast, sleep deterioration at high altitude is primarily triggered by acute hypoxic exposure, with its core physiological feature being frequent micro-arousals and severe sleep fragmentation caused by hypoxic periodic breathing (50). Studies confirm that even in healthy individuals, rapid ascent to high altitude significantly reduces slow-wave sleep and REM sleep (45). Therefore, ΔAIS more likely signifies the intensity of the body’s neuro-respiratory control response to acute hypoxia, rather than merely the experience of “insomnia”. For highly educated individuals, they may simultaneously bear both risks: baseline poor sleep resulting from long-term academic pressure, and the acute stress brought by the high-altitude environment, which superimpose but independently elevate AMS risk. Therefore, intervention strategies should also be separate: improving baseline sleep before embarking for high altitude and implementing targeted measures to ensure sleep quality during the high-altitude stay.

In the multivariate analysis adjusted for age and sex as covariates, this study reached a key conclusion: the effect of baseline AIS scores on AMS is independent of age and sex. This finding differs somewhat from some previous research. For example, Rupert, in a study focusing on a specific mountaineering population, reported significant effects of age and sex (36). However, it must be noted that the existing literature findings on the association between demographic factors and AMS susceptibility are inconsistent, with the magnitude and significance of these effects often varying according to study design, population characteristics, and exposure environment. This does not negate the general risk value of age and sex in broader populations, but rather suggests that within this specific homogeneous group, baseline insomnia symptoms constitute a more prominent and modifiable independent risk marker. Future research with larger and more diverse samples is needed to further delineate the relative contributions and interactions between insomnia symptoms and traditional demographic risk factors in predicting AMS.

This study employed the Athens Insomnia Scale (AIS) to assess subjective sleep quality. It should be noted that, to control for confounding factors, we followed a unified sleep schedule on rest days at base camps. This arrangement may have introduced confounding effects on certain AIS items. For example, ratings for “total sleep time” and “early morning awakening” may have been simultaneously influenced by the behavioral constraints of the fixed schedule and the physiological disturbances of high altitude. Enforced bedtime may alter participants’ perception and reporting of sleep, meaning the AIS scores to some extent reflect adaptation to the schedule rather than solely the specific effects of altitude exposure. Future high-altitude sleep research, if conducted under more flexible sleep schedules or combined with objective sleep monitoring (e.g., actigraphy), would help more clearly disentangle the respective contributions of environmental and behavioral factors to subjective sleep experience.

## 6 Conclusion

The baseline Athens Insomnia Scale (AIS) score remained significantly associated with AMS risk after adjusting for the demographic covariates of age and sex, indicating that the predictive effect of baseline insomnia symptoms on AMS risk is independent of these commonly considered demographic variables. Although the high-altitude AIS score could not be stably estimated in the multivariate model due to complete separation, its strong association in univariate analysis-coupled with the model breakdown itself-suggests this metric possesses considerable predictive power. Therefore, AIS scores (primarily the baseline score) serve as a strong and independent predictor of Acute Mountain Sickness (AMS) in highly educated populations. Furthermore, the influence of baseline and high-altitude insomnia on AMS appears to be independent rather than synergistic, suggesting that targeted sleep interventions can be effectively implemented both during pre-ascent preparation and the high-altitude expedition itself. Based on this study, we recommend incorporating sleep assessment (using standardized tools such as the Athens Insomnia Scale, Pittsburgh Sleep Quality Index, or sleep diaries) into the pre−exposure health screening and risk evaluation system for high−altitude environments (51, 52). This approach holds practical significance for identifying and protecting this highly educated population. Additionally, future research could further explore the dose–response relationship between AIS scores and the severity of acute mountain sickness (AMS), which would provide more precise scientific evidence for developing risk−stratified intervention strategies based on levels of insomnia symptoms.

## 7 Limitations

First, the findings of this study are applicable to scenarios involving “rapid ascent and short-term stays” at altitudes ranging from 2,726 m to 5,396 m. The sample size of this study was relatively small (N = 15), which limited the ability to perform meaningful subgroup analyses or present corresponding descriptive statistics based on different demographic characteristics. Furthermore, the short duration of the mountaineering cycle may not fully capture the complete evolution of symptoms, which may affect the accuracy and generalizability of the results. Second, this study utilized the LLS score from the morning of Day 2 for the primary analysis to focus on the physiological response to initial altitude exposure. Although the mean LLS score was higher after descent on Day 3, this is highly likely a direct consequence of the extreme physical exertion and acute sleep deprivation from the pre-dawn summit push. Therefore, the Day 3 data cannot be simply attributed to prolonged altitude exposure, and this study design cannot disentangle these combined effects. Future studies observing the natural course of AMS should conduct serial assessments on rest days without high-intensity activity interference. Third, regarding the statistical models, we were only able to adjust for basic demographic variables, namely age and sex. Although this is a common approach in studies with limited samples, this strategy does not account for potential residual confounding from other known risk factors for acute mountain sickness, such as individual fitness levels, prior high-altitude exposure history, body mass index, details of ascent rate, and the presence of acute infections. Fourth, the study sample consisted exclusively of highly educated individuals (postgraduate students), and its high homogeneity may limit the generalizability of the results to populations with different educational backgrounds. Fifth, the assessment of sleep in this study relied primarily on the subjective Athens Insomnia Scale. Although the AIS has good validity and is convenient for field administration, the overlapping nature of its assessment periods makes it difficult to precisely attribute changes in insomnia symptoms to specific days following altitude exposure. Future studies could provide more precise temporal evidence for the association between insomnia symptoms and altitude exposure by incorporating daily monitoring methods such as sleep diaries or actigraphy.

## Data Availability

The original contributions presented in the study are included in the article/Supplementary Material. Further inquiries can be directed to the corresponding author.
